# Dietary and Animal Strategies to Reduce the Environmental Impact of Pastoral Dairy Systems Result in Altered Nutraceutical Profiles in Milk

**DOI:** 10.3390/ani12212994

**Published:** 2022-10-31

**Authors:** Cameron Joel Marshall, Konagh Garrett, Stephan Van Vliet, Matthew Raymond Beck, Pablo Gregorini

**Affiliations:** 1 Faculty of Agriculture and Life Sciences, Lincoln University, Lincoln 7647, New Zealand; 2 Department of Nutrition, Dietetics, and Food Sciences, Utah State University, Logan, UT 84322, USA; 3 Livestock Nutrient Management Research Unit, The Agricultural Research Service, The United States Department of Agriculture (USDA-ARS), 300 Simmons Drive, Unit 10, Bushland, TX 79012, USA

**Keywords:** MUNBV, plantain, nutraceutical, metabolomic, milk

## Abstract

**Simple Summary:**

The use of both milk urea nitrogen breeding values and plantain as a diet type have been studied for their ability to reduce the environmental impact of pastoral dairy production practices. This study investigated whether these two techniques also influenced the nutraceutical profile of the milk by investigating the amino and fatty acid as well as the metabolomic profile of milk from cows considered divergent for milk urea nitrogen breed values whilst consuming a diet of either plantain or ryegrass. Both the effect of animal genetics and diet were found to influence the nutraceutical profile of the final product. These results indicate the potential to alter the nutraceutical profile of milk for human consumption to potentially produce healthier products. However, further research is required to determine if the consumption of these milk products will have positive health outcomes for the consumer.

**Abstract:**

The objective of this study was to evaluate and provide further insights into how dairy cows genetically divergent for milk urea N breeding values [MUNBV, high (2.21 ± 0.21) vs. low (−1.16 ± 0.21); µ ± SEM], consuming either fresh cut Plantain (*Plantago lanceolata* L., PL) or Ryegrass (*Lolium perenne* L., RG) herbage, impacted the nutraceutical profile of whole milk by investigating amino and fatty acid composition and applying metabolomic profiling techniques. Both diet and MUNBV, and their interaction term, were found to affect the relative abundance of alanine, glycine, histidine, and phenylalanine in the milk (*p* < 0.05), but their minor absolute differences (up to ~0.13%) would not be considered biologically relevant. Differences were also detected in the fatty acid profile based on MUNBV and diet (*p* < 0.05) with low MUNBV cows having a greater content of total unsaturated fatty acids (+16%) compared to high MUNBV cows and cows consuming PL having greater content of polyunsaturated fatty acids (+92%), omega 3 (+101%) and 6 (+113%) compared to RG. Differences in the metabolomic profile of the milk were also detected for both MUNBV and dietary treatments. Low MUNBV cows were found to have greater abundances of choline phosphate, phosphorylethanolamine, N-acetylglucosamine 1-phosphate, and 2-dimethylaminoethanol (*p* < 0.05). High MUNBV cows had a greater abundance of methionine sulfoxide, malate, 1,5-anhydroglucitol (1,5-AG), glycerate, arabitol/xylitol, 3-hydroxy-3-methylglutarate, 5-hydroxylysine and cystine (*p* < 0.05). Large differences (*p* < 0.05) were also detected as a result of diet with PL diets having greater abundances of the phytochemicals 4-acetylcatechol sulfate, 4-methylcatechol sulfate, and *p*-cresol glucuronide whilst RG diets had greater abundances of 2,6-dihydroxybenzoic acid, 2-acetamidophenol sulfate, and 2-hydroxyhippurate. The results of this study indicate the potential to alter the nutraceutical value of milk from dietary and genetic strategies that have been previously demonstrated to reduce environmental impact.

## 1. Introduction

A growing global population paired with increasing societal concern about environmental externalities from livestock agriculture has spurred demands for more nutritious, healthy, and environmentally friendly products [1,2,3]. Recent research by Marshall et al. [4,5] investigated dietary [use of narrow-leafed plantain (*Plantago lanceolata* L.), PL] and animal genetic-based (use of milk urea nitrogen breeding values; MUNBV) approaches as potential solutions to reduce the environmental impact of pastoral dairy systems. These studies are supported by other work exploring the use of plantain [6,7,8], and selecting for low MUNBV values [9,10] to reduce the environmental impact of pastoral dairy production practices. Marshall and Gregorini [11] hypothesized that the nutritional value of milk for human consumption could differ for milk produced by animals divergent for MUNBV as a result of different ingestive and digestive patterns. 

Oral processing of ingesta through mastication and chewing while ruminating determines the physical characteristics of both ingestive and rumination boli, thereby determining the rate of digestion and digesta outflow from the rumen [12,13]. The greater the oral processing of ingesta and digesta results in smaller particle sizes swallowed [14] resulting in faster digesta outflow from the rumen [15]. Faster digesta outflow rate reduces rumen biohydrogenation rate of fatty acids (FA) [16,17], which in turn increases the supply of polyunsaturated fatty acids (PUFA) to the host animal and its products such as milk [17,18]. Subsequently, factors influencing ingestive and thereby digestive processes, like animal health status, diet, and their interaction [19,20,21,22,23,24], can alter the chemical composition and consequently nutraceutical characteristics of the final product for human consumption [25]. For example, Mangwe et al. [26] reported that cows grazing PL produced milk with a greater content of PUFAs as compared to cows grazing a ryegrass sward [*Lolium perenne* L. (RG)]. Mangwe et al. [26] postulated these results are due to a greater comminution potential and thereby faster digesta passage rate through the rumen in the case of PL feeding. As Marshall et al. [27] reported greater levels of oral processing from grazing dairy cows divergent for MUNBV, it was therefore hypothesized that the resulting faster digesta passage rate may also influence the biological value of the milk for human consumption in a similar manner to what has been observed from dietary manipulation strategies. 

Plants, such as PL, which has been investigated due to its different primary chemistry compared to RG as a mitigation tool to reduce environmental impact has been documented to have an array of secondary bioactive compounds [28,29], that have the potential to (1) reduce oxidative and physiological stress in grazing ruminants [30,31,32], (2) increase animal performance and feed conversion efficiency, thereby reducing environmental impact [33,34], and (3) increase the presence of bioactive compounds with potential nutraceutical, therapeutical, and prophylactic properties in meat and milk [33,35]. 

Therefore, the objective of this study was to evaluate and provide further insights into how dairy cows genetically divergent for MUNBV (high vs. low), consuming either PL or RG fresh herbage, impacted the nutraceutical profile of milk by investigating amino and fatty acid compositions and applying metabolomic profiling techniques.

## 2. Materials and Methods

This study was conducted according to the guidelines of the Lincoln University Animal Ethics Committee (AEC 2019-25A). The methodology of this study has been previously reported in detail [4]. In brief, 16 multiparous lactating Holstein Friesian × Jersey cows were housed in metabolism crates at the Lincoln University Johnstone Memorial Laboratory in Canterbury, New Zealand (43°64′ S, 172°45′ E) from January-February 2020. On average all cows were 150 ± 20.7 (µ ± SEM) days in milk, had an average live weight of 506 ± 35 kg, a body condition score of 3.75 ± 0.25 out of 10, and were 6.7 ± 1.4 years old. Cows were selected from the Lincoln University Ashley Dene Research and Development Station herd based on each cow’s estimated MUNBV (mg/dL) value. Milk urea N breeding values were provided by CRV Ltd. (Hamilton, New Zealand) as part of regular commercial herd testing, with values estimated as per the methodology described by Beatson et al. (2019).

A 2 × 2 factorial design with repeated measurements was implemented over four runs, where cows were assigned either to a whole PL (cv. Agritonic) or RG (cv. One50) fresh cut herbage diet and classified to be in the ‘high’ or ‘low’ MUNBV group based on MUNBV values. Four cows were used per run resulting in one animal per factorial arrangement. A run consisted of 12 days (9 acclimation days and 3 measurement days housed in metabolism crates), with runs conducted concurrently on a three-day delay so that as the first group of cows left the metabolism crates the next entered. The average MUNBV for cows classified as ‘high’ in the PL treatment was 2.19 ± 0.21 and −1.23 ± 0.21 for cows classified as low. Cows in the RG treatment had an average MUNBV of 2.23 ± 0.21 for cows classified as high and −1.09 ± 0.21 for those classified as low. Cows were balanced for production worth, age, and live weight across both dietary treatments and MUNBV groups. 

Before the start of the experiment, all cows were grazing a ryegrass-based sward and were dosed with Rumensin capsules (Elanco, Auckland, New Zealand) one week before the start of the trail to mitigate the risk of bloating. Cows on the PL treatment had the percentage of PL in the diet increased by 20% each day, with a starting level of 20% PL, resulting in a whole diet of PL by day 5. All cows had ad libitum access to fresh drinking water during both the acclimation and measurement period. 

### 2.1. Herbage Measurements

All herbage was cut at ~3 cm above ground level from a standing sward height of ~30 cm daily using a Haldrup (GmbH, Ilshofen, Germany) forage harvester and stored in a 4 °C chiller. Herbage was allocated twice daily at the morning (0700 h) and afternoon (1500 h) milkings. Herbage refusals from the previous period were removed and weighed before fresh herbage was weighed and allocated. Cows were fed with a targeted 10% refusal rate to provide ad libitum access to feed. Samples were taken from each feeding for both allocated and refused herbage to determine dry matter (DM) content as well as chemical and botanical composition. The botanical composition of herbage was determined for both allocated and refused feed by splitting the samples into the representative constituent parts of the allocated herbage type. The DM content of herbage samples as well as botanical composition samples were determined by oven-drying samples for 72 h at 60 °C. Herbage that was analyzed for chemical composition (chemical characteristics, amino acid (AA) and phenolic content) was freeze-dried before being ground in a centrifugal mill (ZM200 Retsch, Haan, Germany) fitted with a 1 mm sieve. 

Ground freeze-dried herbage samples were analyzed for polyphenol content by first using the extraction method of Le et al. [36] before being analyzed using the methodology of Gómez-Alonso et al. [37] on an Agilent high performance liquid chromatograph (HPLC) 2 with quaternary pump and DAD and FLD detectors fitted with an EXL-1110-1546U, ACE 3µ C18-PFP 150 × 4.6 mm column (Advanced Chromatography Technologies, Aberdeen, Scotland, UK). 

Ground freeze-dried herbage samples were assessed for AA concentration using HPLC analysis conducted on a hydrolyzed sample using an Agilent 11,000 series (Agilent Technologies, Walbronn, Germany) fitted with a 150 × 4.6 mm, C18, 3 μm ACE-111–1546 column (Winlab, Scotland, UK) for AA separation. 

Ground freeze-dried herbage was analyzed for organic matter (OM), water-soluble carbohydrates (WSCs), neutral and acid detergent fiber (NDF, ADF), crude protein (CP), and DM, OM, and DM in the OM digestibility (DMD, OMD, DOMD, respectively) values using near-infrared spectrophotometry (NIRS, Model: FOSS NIRSystems 5000, Laurel, MD, USA). The metabolizable energy (ME) content of forage was calculated as per the calculation of the Primary Industries Standing Committee [38] of 0.16 × DOMD. Calibration of NIRS equations was conducted before analysis with all equations having an R^2^ ≥ 0.90.

### 2.2. Animal Measurements

Cows were milked twice daily (0700 and 1500 h), using a portable milking machine (DeLaval vacuum pump DVP170; DeLaval: Tumba, Sweden). The design of the machine allowed for the individual collection of each cow’s milk into separate cisterns. This allowed for the cistern to be weighed after each milking to measure the milk yield (L) per cow at each milking and to take individual milk samples. Milk lines were flushed with 20 L of cold water between cows to avoid any cross-contamination. Representative 100 mL subsamples were taken for analysis of quality attributes (protein, fat, lactose, and somatic cell) using a CombiFoss machine (Foss Electric, Hillerød, Denmark) by MilkTest New Zealand. Milk urea N measurements were determined on skimmed milk using an automated Modular P analyzer [Roche/Hitachi, [39]]. Representative 50 mL subsamples of whole milk were freeze-dried and used for AA analysis using an HPLC Agilent 11,000 series (Agilent Technologies, Walbronn, Germany) fitted with a 150 × 4.6 mm, C18, 3 μm ACE-111–1546 column (Winlab, Market Harborough, Scotland, UK). Freeze-dried whole milk samples were also used to analyze FA methyl esters which were prepared by trans-methylation and assessed through the use of gas chromatography (AOAC method 2012.13) using a Shimadzu GC-2010 gas chromatograph (Shimadzu, Tokyo, Japan).

Metabolomics analysis of whole milk samples was conducted using ultrahigh performance liquid chromatography-tandem mass spectroscopy (UPLC-MS/MS) by Metabolon (Morrisville, North Carolina, USA) as described previously [40]. Briefly, samples were weighed, with recovery standards added for quality control purposes. Proteins were precipitated with methanol under vigorous shaking for 2 min (Glen Mills Genogrinder 2000, Glen Mills, Clifton, NJ, USA)) followed by centrifugation to allow for their removal as well as the recovery of chemically diverse metabolites. Two fractions were analyzed using two reverse phase UPLC-MS/MS with a positive ion mode electrospray ionization (ESI), one analyzed using UPLC-MS/MS with a negative ion mode ESI, another one analyzed using hydrophilic interaction UPLC-MS/MS with a negative ion mode ESI. The UPLC-MS/MS platform utilized a Waters Acquity UPLC with Waters UPLC BEH C18-2.1 × 100 mm, 1.7 μm columns, and a Thermo Scientific Q-Exactive high-resolution/accurate mass spectrometer (ThermoFisher, Waltham, MA, USA) interfaced with a heated electrospray ionization (HESI-II, ThermoFisher, Waltham, MA, USA)) source and Orbitrap mass analyzer (ThermoFisher, Waltham, MA, USA) operated at 35,000 mass resolution. One aliquot was analyzed using acidic, positive ion-optimized conditions, and the other using basic, negative ion-optimized conditions in two independent injections using separate dedicated columns (Waters UPLC BEH C18-2.1 × 100 mm, 1.7 µm). Extracts reconstituted in acidic conditions were gradient eluted using water and methanol containing 0.1% formic acid, while the basic extracts, which also used water/methanol, contained 6.5 mm ammonium bicarbonate. A third aliquot was analyzed via negative ionization following elution from a hydrophilic interaction liquid chromatography (HILIC) column (Waters UPLC BEH Amide 2.1 × 150 mm, 1.7 µm) using a gradient consisting of water and acetonitrile with 10 mm ammonium formate. The MS analysis alternated between MS and data-dependent MS2 scans using dynamic exclusion, and the scan range was from 80–1000 *m*/*z*. Peaks were quantified using area-under-the-curve. A data normalization step was performed to correct variation resulting from instrument inter-day tuning differences by setting the medians to equal one (1.00) and normalizing each data point proportionately (termed the “block correction”). This preserved variation between samples but allowed metabolites of widely different raw peak areas to be compared on a similar graphical scale. Missing values were imputed with the observed minimum after normalization.

### 2.3. Statistical Analysis

All statistical analyses conducted for herbage attributes as well as milk yield and quality attributes were conducted using R [41]. Data were tested for normality using a Shapiro–Wilk’s test to satisfy model assumptions. Normally distributed data were analyzed using a mixed model ANOVA from the ‘lme4′ package and a type III analysis of variance table using the Satterthwaite’s method for determining *p* values. Non-normally distributed data were analyzed using generalized linear mixed model ANOVA and a type II analysis of variance table using Walk Chi-square method for determining *p* values. Model diagnostics were evaluated for all models for the goodness of fit. Residuals were relatively randomly scattered around zero in a residual versus fitted plot; a Q-Q plot of residuals and Cook’s distance indicated the data fit the model well. For milk quality attributes day animal was housed in metabolism crates (1, 2, or 3) was nested within Run (1, 2, 3, or 4) that the cows were used in during the experiment and fitted as random effects for both linear and generalized linear models. 

A linear model based on limma [42] was conducted in MetaboAnalyst 5.0 (https://www.metaboanalyst.ca/ accessed on 10 October 2021) and a ranked heatmap was created of the top 25 metabolites based on Pearson’s distance measurements. Subsequently, metabolites were clustered using ChemRICH (https://chemrich.idsl.me/ accessed on 15 November 2021) software, cluster analysis is conducted via structural similarity and ontology mapping through the use of InChiKeys and SMILES using the ChemRICH identifier list downloaded from (https://chemrich.idsl.me/ accessed on 15 November 2021). Phytochemicals were identified by inputting metabolite Chemical Identifiers (CIDs) into the Indian Medical Plants, Phytochemistry and Therapeutics (IMPPAT) curated database [43], and if metabolites were present in the database they were classified as phytochemicals. Statistical significance was declared at *p* ≤ 0.05 with tendencies discussed at 0.05 < *p* ≤ 0.10. 

## 3. Results

Only results about dietary and milk metabolomic attributes are reported here. Readers are referred to Marshall et al. [4] for milk production, N and water balance results, and Marshall et al. [44] for diel N excretion pattern. Herbage chemical characteristics, botanical composition, and DMI values have been reported previously [4]. In brief, no effect *(p* > 0.05) was detected by diet, MUNBV, or the interaction term on DMI, kg/d. High MUN cows consuming RG had an intake of 15.8 ± 1.19 kg DMI/day, low MUN cows consuming RG had a daily intake of 15.3 ± 1.11, whilst high MUN consuming plantain had an intake of 15.5 ± 1.13 and low MUN cows consuming plantain had a DMI of 14.9 ± 1.09. with cows having on average a DMI of 15.38 ± 1.13 kg/d. Ryegrass diets contained 13 ± 6% weed, 4 ± 0.8% dead material, 3 ± 3% reproductive stem, and 80 ± 4% leaf. The PL diet was comprised of 9 ± 5% weed, 2 ± 1% dead material, 17 ± 3% reproductive stem, and 73 ± 5% leaf. 

Table 1 presents the herbage chemical characteristics as well as the amino acid content for both the offered PL and RG diets. Differences were detected (*p* < 0.05) between the RG and PL forages with RG having a greater DM% (+45%), OM% (+4%), CP% (+11%), NDF% (+70%), ADF% (+9%), OMD% (+2%) and ME content (MJ/kg DM) (+4% g). Meanwhile, PL had a greater WSC content (+19%). Ryegrass had a greater (*p* < 0.05) cysteine content (+54%), asparagine content (+16% greater), alanine content (+19%), taurine content (+290%), lysine content (+29%), proline content (+36%). Plantain was found to have a greater (*p* < 0.05) content of tyrosine (+25%) and methionine (+70%). A tendency (*p >* 0.05) was detected for RG diets to have a greater content of both aspartate and isoleucine content than PL diets.

Table 2 presents the phenolic compound content for the PL and RG diets. Plantain diets (*p* < 0.05) were found to have a greater content of epicatechin (2 times greater), apigenin-7-glucoside (4 times greater), and luteolin (23 times greater). The PL diet also had a greater content of 2,4-hydroxyphenyl ethanol which was not detected in RG samples. Ryegrass diets had a greater (*p* < 0.05) content of chlorogenic acid (5 times greater) as well as a greater content of quercetin and kaempferol both of which had no detected values in the PL diet.

Milk yield parameters have been reported previously [4]. Briefly, an effect of MUNBV (*p* < 0.01) was detected for lactose yield, total solids, and MUN content, with low MUNBV cows having a 9% greater lactose yield and 7% greater total solids (fat and protein) produced compared to high MUNBV cows. High MUNBV cows had an 18% greater MUN content than low MUNBV cows. The only effect of diet (*p* < 0.01) for milk production characteristics was for MUN content with cows consuming PL having a 30% reduction in MUN content compared to cows consuming RG, no effect was detected (*p* > 0.05) for the interaction term between MUNBV and diet. 

Table 3 presents the relative proportions of AA concentrations in milk. An effect of MUNBV (*p* < 0.05) was detected for the relative proportions of methionine, taurine, and valine with low MUNBV cows having a 1%, 10%, and 3% greater relative abundance (respectively) than high MUNBV cows. A diet effect (*p* < 0.05) was detected for the relative abundance of lysine with cows consuming the PL diet having a 2% greater relative abundance compared to cows consuming the RG diet.

Interaction terms were detected between diet and MUNBV for the relative abundances of alanine (*p* < 0.01), glycine (*p* < 0.01), histidine (*p* < 0.05) and phenylalanine (*p* < 0.01). Low MUNBV cows consuming the PL diet had a 4% lower relative abundance of alanine than all other treatments combined. When high MUNBV cows consumed the RG diet the relative abundance of glycine was 2% greater than high MUNBV cows consuming PL and low MUNV cows consuming RG but was not considered different compared to low MUNBV cows consuming PL. Low MUNBV cows consuming the RG diet had a 3% greater relative abundance of histidine compared to all other treatments. High MUNBV cows consuming PL had a 3% greater relative abundance of phenylalanine compared to all other treatments. 

A tendency for an interaction between MUNBV and diet was detected for both arginine (*p* = 0.07) and glutamine (*p* = 0.10). Low MUNBV cows consuming PL had a tendency for a greater relative abundance of arginine compared to all other treatments, whilst high MUNBV cows consuming a PL diet had a tendency for a lower relative abundance of glutamine compared to all other treatments. 

Table 4 presents the individual and summed milk FA composition for cows of high and low MUNBV consuming a diet of either PL or RG. Cows consuming a PL diet were found to have a greater content of omega-3 (*p* < 0.01) (+101%), omega-6 (*p* < 0.01) (+113%) and polyunsaturated FAs (*p* < 0.01) (+92%) compared to cows consuming RG regardless of MUNBV. Cows consuming a diet of RG had a 10% greater content of long chain FA (LCFA) (*p* < 0.01), 23% greater content of total unsaturated FA (TUFA) (*p* < 0.01), and an 8% greater content of total saturated FA (TSFA) (*p* < 0.05) compared to cows consuming PL regardless of MUNBV. Low MUNBV cows were found to have a 16% greater content of TUFA (*p* < 0.01) compared to high MUNBV cows, whilst high MUNBV cows had an 8% greater content of TSFA (*p* < 0.01) compared to low MUNBV cows irrespective of diet. 

An interaction term was detected between diet and MUNBV for the short chain FAs (SCFA) (*p* < 0.05), medium chain FAs (MCFA) (*p* < 0.05), monounsaturated FAs (MUFA) (*p* < 0.05), total branch FAs (*p* < 0.01) and omega 3 to omega 6 ratio (*p* < 0.01). High MUNBV cows consuming PL had a 33% greater SCFA content compared to low MUNBV cows consuming PL but were not considered different than both high and low MUNBV cows consuming RG. High MUNBV cows consuming either PL or RG had a 33% greater MCFA content than low MUNBV cows consuming PL but were considered not different from low MUNBV cows consuming RG (*p* > 0.10). Low MUNBV cows consuming PL had a 34% greater MUFA content than low MUNBV cows consuming RG, as well as both high MUNBV cows consuming PL and RG, all of which were considered not different (*p* > 0.10). Low MUNBV cows consuming RG had an 18% greater total branch chain FA content compared to high MUNBV cows consuming RG, as well as a 52% greater total branch chain FA content compared to cows consuming PL regardless of MUNBV. Low MUNBV cows consuming PL had a 13% lower omega 3 to omega 6 ratios compared to all other treatments, which were considered not different from each other. 

**Figure 1:**
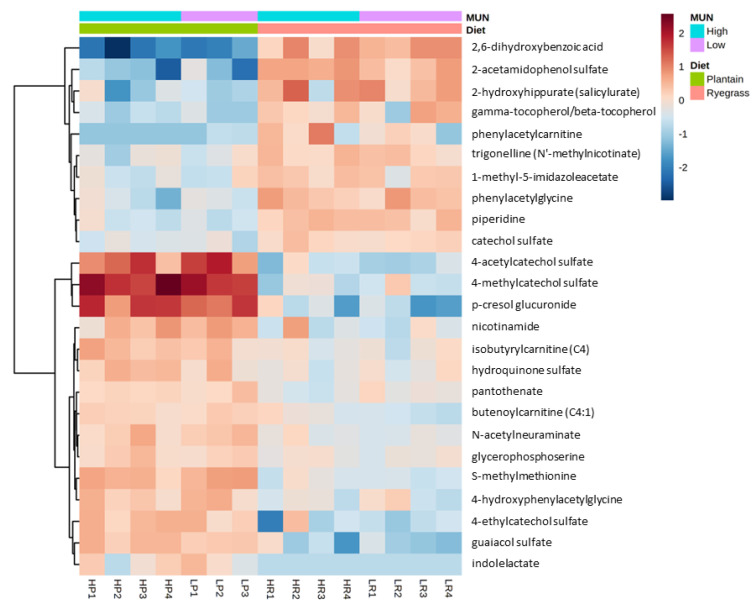
Heatmap of the top 25 metabolites detected in cows milk clustered by a Wards clustering algorithm that are considered statistically different (*p* < 0.05) based on a linear model where diet (plantain or ryegrass) was considered the primary factor and animal genetics (milk urea nitrogen breeding values) was considered a covariate. A red colour indicates a higher abundance of the corresponding metabolite and a blue colour indicates a lower abundance of the corresponding metabolite.

Figure 1 presents a heatmap of the top 25 metabolites ranked by *p* value that were considered different (*p* < 0.05) based on the diet treatment factor with MUNBV as a co-variate. 

Table 5 presents the metabolites that were found to have the greatest difference by diet as indicated by the results in Figure 1 and identified using the Indian Medical Plants, Phytochemistry, and Therapeutics (IMPPAT) curated database. The milk from the PL diet was found to have a greater content of detected phytochemicals compared to the RG diet, with eight of the detected phytochemicals being present in greater quantities compared to the three detected for RG.

Table 6 presents metabolites that were found to differ (*p* < 0.05) by MUNBV when the diet effect was included as a covariate. Low MUNBV cows had a greater abundance of choline phosphate, phosphorylethanolamine, N-acetyl-glucosamine 1-phosphate, and 2-dimethylaminoethanol. High MUNBV cows had a greater abundance of methionine sulfoxide, malate, 1,5-anhydroglucitol (1,5-AG), glycerate, arabitol/xylitol, 3-hydroxy-3-methylglutarate, 5-hydroxylysine, and cystine. 

Table 7 presents the metabolite classes identified to be altered [*p* < 0.01, False Discovery Rate (FDR) < 0.05] between a PL and an RG-based diet, with PL-based diet used as the reference point. ChemRICH analysis indicates that the most significant (FDR ≤ 0.01) metabolite class that was upregulated on a PL diet compared to an RG diet is the metabolites relating to the benzoate metabolism with the most affected metabolite in this class being 4-methylcatechol sulfate. 

## 4. Discussion

In this study, we aimed to evaluate and provide further insights into how dairy cows genetically divergent for MUNBV (high vs. low), consuming either PL or RG fresh-cut herbage, impacted nutraceutical profiles of milk by investigating amino and fatty acid compositions and applying metabolomic profiling techniques. Several differences were found in the milk AA and FA content, as well as the metabolomic profile as a result of genetic and dietary factors, indicating the potential for genetic and dietary manipulations to influence the nutraceutical value of milk, in addition to environmental properties as previously explored [4]. 

The differences in the primary chemistry, AA content, and phenolic compounds in the RG and PL diet used in this study are potentially explanatory factors for the variation in milk composition between the two diets. The different nutrient supply from the two diets may have facilitated different ruminal fermentation, nutrient availability, and thereby nutrient absorption from the animal and thus altering the final composition of the milk. The differences between the FA composition between the RG and PL diets are likely explained by different rates of ruminal biohydrogenation and FA composition of the forages. Plantain is known to be easily masticated and therefore is likely to have a relatively smaller ingested particle size relative to the RG diet [45]. Smaller particle sizes can be associated with a greater passage rate from the rumen [46] as a result of greater surface area for microbial activity [47]. In turn, the associated size reduction will facilitate escape from the rumen, with a faster passage rate being associated with less exposure of dietary lipids to biohydrogenation in the diet [48]. Therefore, it could be assumed that the greater content of total unsaturated fatty acids in the milk from cows consuming the PL diet is a result of a faster rumen digesta flow rate and associated reduction in biohydrogenation. This is in line with other studies suggesting a similar relationship when comparing a PL diet to an RG white clover diet [26]. 

The greater content of TUFA based on MUNBV could also be indicative of reduced ruminal biohydrogenation. Cows with low MUNBV across both diets had a greater TUFA content than high MUNBV cows. Marshall et al. [4] hypothesized that low MUNBV cows may have a faster digesta outflow rate due to the differences in mastication behavior [27], which may have also resulted in less time for biohydrogenation to occur in the rumen resulting in low MUNBV cows having greater TUFA than high MUNBV cows. The greater content of unsaturated fatty acids in milk from both cows consuming PL and low MUNBV cows could be considered to represent a potentially greater nutraceutical value. Prospective studies have indicated that replacing saturated fatty acids with unsaturated fatty acids can reduce the risk of cardiovascular disease in humans [49,50]. Although more long-term studies are likely required, it could be considered beneficial to have milk from cows consuming PL and from low MUNBV due to their relatively higher content of unsaturated fatty acids in terms of human health. 

The differences in the amino acid composition in the milk may also be related to differences in digesta fermentation differences. It could be speculated that the different plant secondary compounds from the PL [51] diet may be altering the relative abundance of different microbes in the rumen, with differences in microbe relative abundance between an RG-based diet and PL report previously [52]. The combination of different microbe species’ relative abundance and interaction with PSC may result in an altered AA absorption in the rumen, thus influencing the flow of AA to the duodenum of the animal which in turn will influence the AA composition of the milk. Smaller particle sizes have been associated with lower abundances of protozoa [53], which in turn have been associated with greater efficiency of microbial N incorporation [46,54]. It could also be considered that difference in particle size of ingesta and digesta of PL compared to RG may have influenced microbial yield and therefore AA supply to the duodenum, as faster digesta outflows have been reported to change rumen fermentation towards a more glucogenic pattern [55] and increase microbial growth rate and yield by differentiating microbial population [56]. A similar mechanism may also be responsible for the differences observed in the milk AA composition based on MUNBV. Animals divergent for MUNBV have been documented to have differences in the relative abundance of microbes in the rumen [57]. Consequently, it is postulated that, because of different rumen functions, abundance, and composition of rumen microorganisms, a different ratio of AA may be supplied to the host animal by the rumen, resulting in different AA compositions in the milk. A differing supply of AA may also affect the health status of the animal [58], however, no differences in health status were recorded or observed during this study. However, further studies are required to understand this relationship in the context of dairy cows differing in MUNBV. Moreover, the absolute difference in amino acids between animals divergent for MUNBV and differing in provided diet (RG vs. PL) is considered small (Table 3) and we question its biological relevance. 

Several differences were detected in the metabolic profile of the milk, with some of the largest differences being the greater content of 4-acetylcatechol sulfate, 4-methcatechol sulfate, and *p*-cresol glucuronide in the PL diet compared to the RG diet. These findings are likely related to the greater abundance of the phenolics epicatechin, luteolin, and/or apigenin-7-glucoside in the PL forage, as several of these phenolic metabolites found in milk are potential downstream metabolites of the aforementioned phenolics. The greater content of 4-acetylcatechol could be of interest to human nutrition due to its ability to reduce prostaglandin E_2_ [59], which is an inflammatory mediator. An in vivo study in mice determined that 4-methcatechol sulphate could act as a vaso-relaxer [60], while other studies have indicated its antiplatelet effect [61] and the ability to induce apoptotic death of murine tumor cells [62]. On the other hand, cows consuming the RG diet had a greater content of 2,6 dihydroxybenzoic acid which is an anti-oxidant with the potential to have beneficial health effects [63,64] and 2-hydroxyhippurate which has been associated with chemoprevention [65], thus indicating both diets have the potential to provide beneficial compounds for animal health. The differences observed in the metabolomic profile in this study and others indicates [66,67,68] the possibility to alter the nutraceutical value of the final product and thus presents an opportunity for designing dietary strategies for ruminants to potentially target health outcomes for the final consumer. 

Differences were also detected in the metabolomic profile in milk based on MUNBV. The cause of these differences is unknown but could be related to the different ingestion and digestion dynamics from MUNBV reported previously [4,27]). Whilst the effect of MUNBV on metabolomic differences in milk was small relative to diet, its presence indicates the possibility for animal-based solutions to be implemented by breeding for a more beneficial/healthier milk composition. For example, the greater content of phosphorylethanolamine in the milk of low MUNBV cows could be associated with alleviated mitochondrial stress responses from caffeine ingestion as has been documented in *Caenorhabditis elgan* models [69]. 

Whilst the primary objective of this study was to investigate the ability to alter the nutraceutical value of milk from the use of MUNBV and different diets, the data collected also allows some inferences on the health and well-being of the animals which produce the product. Both forages in this study contain phenolic compounds with known beneficial health benefits in different quantities. For example, the RG diet contained a greater quantity of chlorogenic acid compared to the PL diet. Chlorogenic acid has known antioxidant, immunoprotective, and anti-inflammatory characteristics [70,71,72] and has been investigated for anticarcinogenic properties in humans and mice [73]. The PL diet contained greater; epicatechin which has known antitumor properties [74], apigenin-7-glucoside, with the apigenin conjugates being considered to have potent antioxidant and anti-inflammatory effects [75], as well as greater luteolin content with known antioxidant and anti-inflammatory effects [76]. Whilst studies have explored these compounds mainly in alternate animal models, it is plausible that the presence of such compounds in the diets of cattle, will also provide benefits for the cattle, however, future research is required to confirm this. 

If the presence of these compounds in dairy has an appreciable impact on consumer health responses to its consumption is currently not known and requires further human nutrition studies. It should be considered that the bioavailability and utilization pathways of these individual metabolites are not well documented or specifically investigated from the consumption of bovine milk. It is therefore only speculative as to any beneficial human health outcomes that may occur as a result of the ingestion of any of these products. However, the ability to alter the composition of these metabolites indicates the future potential for strategic breeding and diet planning to potentially incorporate healthier nutraceutical properties into animal-based dairy products. Future research will be required to determine the concentrations and magnitude of differences in these compounds that would be considered biologically significant for animal health and the end consumer.

Differences in the metabolomic profiles of milk produced by cows within this study suggest the potential for the use of metabolomic analysis to allow for inferences on the health of the animal producing it. For example, the presence of 1,5-anhydroglucitol (1,5-AG) in a greater concentration in the metabolomic profile of the PL diet milk could be seen as a potential indicator of the metabolic health of the animal. 1,5-anhydroglucitol is used as a glycemic marker in humans [77] with its level measured in blood serum used as an index of the degree of urinary glucose excretion [78] which can allow for inferences regarding the health status of the individual. The ability to detect 1,5-AG in milk may present an area of future research where additional metabolomic markers may be able to be utilized to determine the metabolic health of the animal. Further research is required to understand the relationship between health and welfare status and the detection of these metabolomic markers. 

Both diets investigated in this study were documented to show differing levels of beneficial compounds. These results highlight that no one specific diet is likely to provide a balance nutritive profile to a grazing animal, and therefore monotonous diets may be deleterious relative to a more taxonomically diverse diet provided in a functional manner which may provide a greater range of available phytonutrients allowing for grazing animals to better meet their needs and self-medicate [79,80]. Previous literature also indicates that animals grazing more diverse forages typically also have a more diverse phytochemical profile in their meat and milk as different forages provide different compounds [35]. It could therefore be considered that the use of diverse diets containing multiple plants may further alter the nutraceutical profile of the end product. It should be considered that the results from a grazing experiment may differ from the results of this experiment where herbage was cut and transported in the morning, thus removing any temporal variation in primary and secondary plant chemical compounds that a grazing animal may be exposed to. 

## 5. Conclusions

Differences were detected in the composition of whole raw milk as a result of diet (ryegrass vs. plantain) and milk urea nitrogen breeding values. Both diets differed in primary and secondary chemistry characteristics resulting in distinct differences in the nutraceutical profile of the milk, particularly in phytochemicals with potential differences in health and flavor properties. Cows consuming the PL diet had greater 4-acetylecatechol content, whilst cows consuming the RG diet had higher 2,6 dihydroxybenzoic acid content. These compounds are broadly classified as having potential anti-oxidant and anti-inflammatory effects. Low MUNBV cows were also found to have a greater content of phosphorylethanolamine, which also could be associated as beneficial for human consumption due to its role in reducing mitochondrial stress. The findings of this study demonstrate the potential to alter the nutraceutical profile of milk for human consumption. These findings, therefore, present the opportunity for strategic dietary management practices as well as the use of animal-based solutions as tools to produce animal-based products with higher nutraceutical value. The use of both low MUNBV and PL diets has previously been implemented to reduce the environmental impact of pastoral dairy production practices, this study shows that not only are these helpful tools for reducing the environmental impact but also potentially an opportunity for a higher quality final product. Further research is required to fully understand if the detected differences in metabolites in milk and their ascribed health benefits will augment human health outcomes after consumption.

## Figures and Tables

**Table 1:** Herbage chemical characteristics and amino acid content of feed provided to cows considered divergent for milk urea nitrogen breeding values (MUNBV) consuming a diet of either plantain or ryegrass.

Item ^1^	Plantain	Ryegrass	SE	*p*-Value
Quality				
DM, %	12.8	18.5	0.71	<0.01
OM, %	87.7	90.9	0.36	<0.01
CP, %	13.4	14.9	0.65	0.01
WSC, %	19.1	16	1.19	0.02
NDF, %	27.3	46.5	0.59	<0.01
ADF, %	24.1	26.2	0.35	<0.01
DMD, %	74.0	75.1	0.61	0.15
OMD, %	77.6	79.5	0.76	0.04
ME, MJ/kg DM	11.1	11.5	0.10	<0.01
Amino acids (g/kg DM)				
Aspartate	7.89	9.35	0.52	0.07
Glutamate	7.25	6.89	0.52	0.39
Cysteine	1.63	2.51	0.47	<0.01
Asparagine	0.37	0.43	0.10	<0.01
Serine	3.80	3.97	0.37	0.46
Histidine	2.68	2.85	0.60	0.41
Glycine	5.37	5.74	0.54	0.35
Threonine	3.99	4.12	0.22	0.60
Arginine	5.80	6.01	0.54	0.61
Alanine	5.18	6.17	0.30	0.02
Taurine	0.21	0.82	0.20	<0.01
Tyrosine	2.38	1.91	0.31	<0.01
Valine	4.15	4.33	0.31	0.49
Methionine	1.21	0.71	0.24	<0.01
Phenylalanine	4.71	4.65	0.22	0.84
Isoleucine	3.54	3.16	0.42	0.07
Lysine	3.58	4.63	0.61	<0.01
Leucine	6.97	6.85	0.44	0.77
Proline	5.00	6.81	1.24	<0.01

^1^ DM, Dry Matter; OM, Organic Matter; CP, Crude Protein; WSC, Water Soluble Carbohydrates; NDF, Neutral Detergent Fibre; ADF, Acid Detergent Fibre; DMD, Dry Matter Digestibility; OMD, Organic Matter Digestibility; ME, Metabolizable Energy.

**Table 2:** Herbage phenolic compound content of feed provided to cows considered divergent for milk urea nitrogen breeding values (MUNBV) consuming a diet of either plantain or ryegrass.

Item (mg/kg DM)	Plantain	Ryegrass	SE	*p*-Value
Gallic acid	12.28	12.05	3.17	0.96
Protocatechuic acid	15.66	24.36	3.83	0.13
2,4-Hydroxyphenyl ethanol	4.86	N.D. ^1^	0.45	
Procyanidin B2	6.24	3.62	1.79	0.32
Chlorogenic acid	637.27	2982.79	960.54	<0.01
Catechin	27.78	8.78	12.71	0.25
Procyanidin B1	10.06	5.16	3.42	0.20
Caffeic acid	5.07	10.31	2.16	0.10
Epicatechin	16.59	8.27	2.79	0.05
Quercetin-3-glucoside	32.97	44.88	8.57	0.34
Apigenin-7-glucoside	78.26	21.06	7.91	<0.01
Luteolin	104.86	4.60	12.30	<0.01
Quercetin	N.D.	2.53	0.52	
Apigenin	9.35	16.28	3.68	0.07
Diosmetin	13.32	12.32	4.14	0.79
Kaempferol	N.D.	3.22	0.51	

^1^ Not Detected, values for these compounds were either not present in the sample or were below the detection limits for the analysis conducted.

**Table 3:** The relative proportion of milk amino acid for cows considered divergent for milk urea nitrogen breeding values (MUNBV) consuming a diet of either plantain (PL) or ryegrass (RG).

Item	High MUNBV			Low MUNBV			*p*-Value		
	PL	RG	SE	PL	RG	SE	MUNBV	Diet	MUNBV × Diet
Alanine	2.84 ^b^	2.96 ^a^	0.04	2.97 ^a^	2.95 ^a^	0.04	<0.01	<0.01	<0.01
Arginine	3.76	3.81	0.05	3.87	3.80	0.05	0.19	0.89	0.07
Aspartate	4.73	4.7	0.34	4.67	4.65	0.33	0.34	0.68	0.31
Cysteine	3.98	3.95	0.52	3.98	3.96	0.53	0.96	0.74	0.75
Glutamine	0.044	0.046	0.003	0.05	0.046	0.045	0.32	0.45	0.10
Glutamic acid	19.5	19.6	0.51	18.7	18.8	0.51	0.08	0.83	0.51
Glycine	1.71 ^b^	1.75 ^a^	0.04	1.74 ^ab^	1.70 ^b^	0.04	0.20	0.60	<0.01
Histidine	3.38 ^b^	3.35 ^b^	0.06	3.39 ^b^	3.46 ^a^	0.06	0.02	0.75	0.02
Isoleucine	4.62	4.63	0.08	4.59	4.6	0.08	0.36	0.74	0.38
Leucine	9.01	8.97	0.16	8.93	8.9	0.16	0.12	0.43	0.32
Lysine	9.69	9.52	0.45	9.89	9.71	0.47	0.12	0.03	0.87
Methionine	2.25	2.23	0.13	2.33	2.31	0.14	0.03	0.49	0.53
Phenylalanine	4.43 ^a^	4.34 ^b^	0.05	4.30 ^b^	4.33 ^b^	0.05	<0.01	0.03	<0.01
Proline	9.87	10.1	0.27	9.94	10.18	0.28	0.73	0.27	0.15
Serine	5.52	5.46	0.18	5.46	5.4	0.18	0.34	0.37	0.99
Taurine	0.16	0.15	0.02	0.17	0.17	0.02	0.01	0.55	0.51
Threonine	4.11	4.15	0.06	4.12	4.16	0.06	0.76	0.13	0.11
Tyrosine	4.03	4.02	0.04	4.00	4.00	0.04	0.27	0.65	0.14
Valine	5.00	5.00	0.011	5.17	5.17	0.12	0.01	0.93	0.49

Differing superscripts between rows indicates a significant difference (*p* < 0.05) based on the interaction term.

**Table 4:** Milk fatty acids composition (g/100 g) for cows considered divergent for milk urea nitrogen breeding values (MUNBV) consuming a diet of either plantain (PL) or ryegrass (RG).

Item ^1^	High MUNBV			Low MUNBV			*p*-Value		
	PL	RG	SE	PL	RG	SE	MUNBV	Diet	MUNBV × Diet
C4:0	1.54	1.50	0.06	1.50	1.46	0.07	0.57	0.48	0.21
C6:0	1.50 ^a^	1.29 ^b^	0.06	1.24 ^b^	1.30 ^b^	0.07	0.05	0.23	0.04
C8:0	1.03 ^a^	0.82 ^b^	0.05	0.77 ^b^	0.81 ^b^	0.06	0.02	0.10	0.03
C10:0	2.66 ^a^	1.95 ^b^	0.16	1.74 ^b^	1.88 ^b^	0.19	0.01	0.12	0.03
C10:1	0.20	0.18	0.02	0.17	0.16	0.02	0.33	0.61	0.15
C12:0	3.04 ^a^	2.28 ^b^	0.19	1.96 ^b^	2.20 ^b^	0.23	0.01	0.21	0.03
C13:0 anteiso	0.06	0.06	0.01	0.05	0.05	0.01	0.48	0.81	0.23
C13:0	0.07 ^a^	0.05 ^ab^	0.005	0.04 ^b^	0.05 ^ab^	0.007	0.06	0.76	0.04
C14:0 iso	0.08 ^b^	0.10 ^a^	0.006	0.06 ^b^	0.11 ^a^	0.007	0.55	<0.01	0.04
C14:0	10.51 ^a^	10.00 ^a^	0.52	7.69 ^b^	9.49 ^ab^	0.62	<0.01	0.25	0.05
C15:0 iso	0.20 ^b^	0.30 ^a^	0.02	0.15 ^c^	0.32 ^a^	0.02	0.77	<0.01	<0.01
C14:1 c9	0.58	0.67	0.06	0.58	0.68	0.07	0.96	0.24	0.54
C15:0 anteiso	0.44 ^b^	0.50 ^b^	0.03	0.30 ^c^	0.60 ^a^	0.04	0.52	<0.01	<0.01
C15:0	1.18 ^a^	1.15 ^a^	0.05	0.92 ^b^	1.26 ^a^	0.06	0.13	<0.01	<0.01
C16:0 iso	0.23 ^bc^	0.25 ^b^	0.10	0.21 ^c^	0.29 ^a^	0.01	0.43	<0.01	<0.01
C16:0	30.0	33.7	0.96	28.3	32.0	1.08	0.16	<0.01	0.30
C16:1 t9	0.09	0.16	0.02	0.10	0.17	0.02	0.72	<0.01	0.39
C16:1 c7	0.31	0.22	0.01	0.32	0.24	0.01	0.23	<0.01	0.63
C16:1 c9	1.18 ^b^	1.38 ^b^	0.10	1.76 ^a^	1.34 ^b^	0.13	0.02	0.30	0.01
C17:0 iso	0.45	0.51	0.03	0.52	0.59	0.03	0.05	0.06	0.44
C17:0 anteiso	0.65 ^a^	0.59 ^b^	0.02	0.58 ^b^	0.69 ^a^	0.02	0.35	0.26	<0.01
C17:0	0.70	0.73	0.03	0.76	0.80	0.03	0.05	0.27	0.92
C17:1	0.34 ^b^	0.35 ^b^	0.03	0.57 ^a^	0.41 ^b^	0.04	<0.01	0.04	0.03
C18:0 iso	0.12	0.06	0.01	0.13	0.07	0.01	0.18	<0.01	0.78
C18:0	11.3	11.9	0.78	11.3	12.0	0.88	0.98	0.52	0.22
C18:1 t5-8	0.14	0.16	0.01	0.13	0.15	0.01	0.14	0.03	0.09
C18:1 t9	0.14	0.15	0.01	0.15	0.15	0.01	0.24	0.75	0.18
C18:1 t10	0.21	0.18	0.01	0.19	0.16	0.01	0.36	0.04	0.06
C18: t11	1.07	2.49	0.23	1.21	2.63	0.26	0.57	<0.01	0.21
C18:1 c6	0.39	0.31	0.03	0.37	0.29	0.03	0.16	<0.01	0.21
C18:1 c9	21.1	18.6	1.22	25.6	22.1	1.74	<0.01	0.05	0.06
C18:1 t15/c10	0.40	0.26	0.02	0.38	0.25	0.03	0.41	<0.01	0.45
C18:1 c11	0.47 ^b^	0.50 ^b^	0.05	0.75 ^a^	0.50 ^b^	0.05	0.01	0.04	0.01
C18:1 c12	0.13	0.07	0.006	0.14	0.08	0.006	0.12	<0.01	0.60
C18:1 c13	0.09 ^b^	0.10 ^b^	0.01	0.15 ^a^	0.10 ^b^	0.01	0.01	0.14	0.01
C18:1 c14/t16	0.58	0.46	0.04	0.53	0.41	0.04	0.12	<0.01	0.19
C18:2 t9,12	0.08	0.12	0.01	0.09	0.12	0.01	0.43	<0.01	0.97
C18:2 c9 t13	0.22	0.12	0.01	0.23	0.13	0.01	0.65	<0.01	0.32
C18:2 c9 t12	0.35	0.25	0.03	0.36	0.26	0.03	0.78	<0.01	0.43
C18:2 t9 c12	0.16	0.11	0.01	0.17	0.11	0.01	0.80	<0.01	0.29
C19:0	0.11	0.11	0.02	0.11	0.11	0.02	0.67	0.99	0.49
C18:2 c9,12	2.17	1.00	0.12	2.22	1.01	0.13	0.62	<0.01	0.64
C18:3 c9,12,15	2.31	1.05	0.12	2.16	1.02	0.12	0.19	<0.01	0.11
C20:0	0.16 ^a^	0.13 ^bc^	0.01	0.12 ^c^	0.15 ^ab^	0.01	0.16	0.81	<0.01
CLA c9 t11	0.53	0.95	0.08	0.62	1.04	0.09	0.37	<0.01	0.92
C20:1 c8	0.03	0.06	0.01	0.02	0.06	0.01	0.66	<0.01	0.53
C20:1 c9	0.11	0.10	0.01	0.11	0.10	0.01	0.97	0.68	0.19
C20:1 c11	0.05	0.04	0.005	0.06	0.05	0.005	0.21	0.05	0.19
C20:3 c8,11,14	0.067 ^a^	0.039 ^b^	0.006	0.041 ^b^	0.039 ^b^	0.004	0.01	<0.01	0.04
C20:4 c5,8,11,14	0.08	0.06	0.005	0.06	0.05	0.005	0.04	0.02	0.25
C22:0	0.088 ^a^	0.074 ^bc^	0.01	0.066 ^c^	0.084 ^ab^	0.01	0.41	0.96	<0.01
C22:1 c13	0.07	0.06	0.01	0.05	0.05	0.01	0.11	0.67	0.06
C20:5 c5,8,11,14,17	0.13 ^a^	0.08 ^b^	0.01	0.08 ^b^	0.08 ^b^	0.01	0.02	0.03	0.05
C23:0	0.07 ^a^	0.04 ^c^	0.009	0.06 ^ab^	0.05 ^bc^	0.007	0.53	<0.01	0.04
C24:0	0.04	0.05	0.005	0.04	0.05	0.005	0.84	0.25	0.08
C22:5 c7,10,13,16,19	0.16	0.14	0.02	0.14	0.11	0.02	0.16	0.15	0.28
Summed									
SCFA	4.11 ^a^	3.57 ^ab^	0.26	3.08 ^b^	3.61 ^ab^	0.22	0.07	0.79	0.02
MCFA	16.2 ^a^	14.2 ^a^	0.92	11.4 ^b^	13.6 ^ab^	0.77	<0.01	0.91	0.01
LCFA	43.5	48.0	0.88	41.8	46.2	0.99	0.12	<0.01	0.85
Omega3	2.61	1.27	0.11	2.40	1.22	.011	0.10	<0.01	0.16
Omega6	2.35	1.10	0.13	2.34	1.10	0.14	0.95	<0.01	0.63
MUFA	23.1 ^b^	23.1 ^b^	1.30	31.4 ^a^	24.3 ^b^	2.04	<0.01	0.03	0.04
PUFA	5.75	2.97	0.26	5.56	2.92	0.26	0.35	<0.01	0.72
Total branch chain FA	1.09 ^c^	1.34 ^b^	0.05	0.99 ^c^	1.58 ^a^	0.05	0.09	<0.01	<0.01
TUFA	30.1	24.9	1.43	35.5	28.5	1.96	<0.01	<0.01	0.09
TSFA	64.0	65.7	1.75	56.2	63.6	1.72	<0.01	0.02	0.06
O3 to O6 ratio	1.12 ^a^	1.11 ^a^	0.05	0.97 ^b^	1.14 ^a^	0.05	0.03	<0.01	<0.01

Differing superscripts between rows indicate a significant difference (*p* < 0.05) based on the interaction term. ^1^ SCFA, short chain fatty acids; MCFA, medium chain fatty acid; LCFA, long chain fatty acid; MUFA, monounsaturated fatty acids; PUFA, polyunsaturated fatty acids; TUFA, total unsaturated fatty acids; TSFA, total saturated fatty acids.

**Table 5:** Metabolites that were detected in the milk of cows consuming plantain and ryegrass with genetic merit (milk urea nitrogen breeding values) controlled for as a covariate that was classified as phytochemical metabolites using the Indian Medical Plants, Phytochemistry and Therapeutics (IMPPAT) curated database. Log fold change (LogFC) values are calculated with the PL diet as the reference group.

Super Pathway	Sub Pathway	Biochemical Name	^1^ CAS ID	LogFC	*p*-Value
Amino Acid	Lysine Metabolism	Pipecolate	4043-87-2	−0.28	0.03
	Methionine, Cysteine, ^2^ SAM, and Taurine Metabolism	S-methylcysteine	1187-84-4	−0.35	0.04
Carbohydrates	Glycolysis, Gluconeogenesis, and Pyruvate Metabolism	Glucose	50-99-7	−0.19	<0.05
		1,5-anhydroglucitol (1,5-AG)	154-58-5	−0.32	0.03
Cofactors and Vitamins	Nicotinate and Nicotinamide Metabolism	Trigonelline (N’-methylnicotinate)	535-83-1	0.61	<0.01
		Nicotinamide	98-92-0	−0.81	<0.01
	Tocopherol Metabolism	Alpha-tocopherol	59-02-9; 10,191-41-0	−0.36	0.02
Xenobiotics	Food Component/Plant	Piperidine	110-89-4	0.85	<0.01
	Benzoate Metabolism	Catechol sulfate	4918-96-1	0.55	<0.01
		*p*-cresol sulfate	3233-57-7	−0.29	0.05
Lipid	Ketone Bodies	3-hydroxybutyrate (BHBA)	625-72-9	−0.43	0.03

^1^ CAS ID, Chemical Abstracts Service Identifier; ^2^ SAM, S-adenosylmethionine.

**Table 6:** Metabolites detected in the milk of cows considered divergent for milk urea nitrogen breeding values (MUNBV) and classified as either high or low when the diet effect (plantain or ryegrass) was controlled for. Log fold change (Log FC) values are calculated with the high MUNBV group as the reference group.

Super Pathway	Sub Pathway	Biochemical Name	^1^ CAS ID	Log FC	*p*-Value
Amino Acid	Lysine Metabolism	5-hydroxylysine	13204-98-3	−0.61	0.03
	Methionine, Cysteine, SAM, and Taurine Metabolism	Cystine	56-89-3	−0.96	0.03
		Methionine sulfoxide	3226-65-1	−0.28	0.05
Carbohydrate	Glycolysis, Gluconeogenesis, and Pyruvate Metabolism	1,5-anhydroglucitol (1,5-AG)	154-58-5	−0.33	0.02
		Glycerate	600-19-1	−0.41	0.02
	Pentose Metabolism	Arabitol/xylitol	-	−0.47	0.04
	Aminosugar Metabolism	N-acetyl-glucosamine 1-phoshate	31281-59-1	1.41	0.02
Energy	^2^ TCA Cycle	Malate	6915-15-7	−0.32	0.03
Lipid	Phospholipid Metabolism	Choline phosphate	72556-74-2	1.93	<0.01
		Phosphorylethanolamine	1071-23-4	1.47	<0.01
	Mevalonate Metabolism	3-hydroxy-3-methylglutarate	503-49-1	−0.48	<0.01
Xenobiotics	Food Component/Plant	2-dimethylaminoethanol	108-01-0	0.40	0.02

^1^ CAS ID, Chemical Abstracts Service Identifier; ^2^ TCA, Tricarboxylic acid.

**Table 7:** Metabolite class identified using chemical similarity enrichment analysis software (ChemRICH https://chemrich.idsl.me/home, accessed on 25 September 2022) for metabolites detected in milk from cows consuming either a plantain or ryegrass diet, plantain is used as the reference diet for this analysis.

Cluster Name	Cluster Size	*p*-Value	FDR ^1^	Key Compound	CAS ID	Altered Metabolites	Greater in Plantain	Greater in Ryegrass	Increased Ratio	Altered Ratio
Benzoate Metabolism	19	<0.01	<0.01	4-methylcatechol sulfate		14	8	3	0.7	0.7
Acetylated Peptides	3	<0.01	<0.01	Phenylacetylglycine	500-98-1	3	1	2	0.3	1
Fatty Acid Metabolism	4	<0.01	<0.01	Butyrylglycine	20208-73-5	3	2	0	1	0.8
Primary Bile Acid Metabolism	3	<0.01	<0.01	Glycocholate	475-31-0, 863-57-0	3	0	1	0.3	1
Long Chain Polyunsaturated Fatty Acid (n3 and n6)	9	<0.01	0.01	Linolenate [alpha or gamma; (18:3n3 or 6)]		1	0	0	1	0.1
Food Component/Plant	18	<0.01	0.02	Piperidine	110-89-4	7	1	3	0.4	0.4
Pyrimidine Metabolism, Orotate containing	4	<0.01	0.02	N-carbamoylaspartate	923-37-5	3	1	0	1	0.8
Lysine Metabolism	10	<0.01	0.04	Pipecolate	4043-87-2	2	2	0	1	0.2

^1^ FDR, False Discovery Rate.

## Data Availability

Data is available upon request.
